# Voltage sensor current, SR Ca^2+^ release, and Ca^2+^ channel current during trains of action potential‐like depolarizations of skeletal muscle fibers

**DOI:** 10.14814/phy2.15675

**Published:** 2023-05-05

**Authors:** Hugo Bibollet, Elton L. Nguyen, Daniel R. Miranda, Christopher W. Ward, Andrew A. Voss, Martin F. Schneider, Erick O. Hernández‐Ochoa

**Affiliations:** ^1^ Department of Biochemistry and Molecular Biology University of Maryland School of Medicine Baltimore Maryland USA; ^2^ Department of Biological Sciences Wright State University Dayton Ohio USA; ^3^ Department of Orthopedics University of Maryland School of Medicine Baltimore Maryland USA

## Abstract

In skeletal muscle, Ca_*V*_1.1 serves as the voltage sensor for both excitation‐contraction coupling (ECC) and L‐type Ca^2+^ channel activation. We have recently adapted the technique of action potential (AP) voltage clamp (APVC) to monitor the current generated by the movement of intramembrane voltage sensors (I_Q_) during single imposed transverse tubular AP‐like depolarization waveforms (I_QAP_). We now extend this procedure to monitoring I_QAP_, and Ca^2+^ currents during trains of tubular AP‐like waveforms in adult murine skeletal muscle fibers, and compare them with the trajectories of APs and AP‐induced Ca^2+^ release measured in other fibers using field stimulation and optical probes. The AP waveform remains relatively constant during brief trains (<1 sec) for propagating APs in non‐V clamped fibers. Trains of 10 AP‐like depolarizations at 10 Hz (900 ms), 50 Hz (180 ms), or 100 Hz (90 ms) did not alter I_QAP_ amplitude or kinetics, consistent with previous findings in isolated muscle fibers where negligible charge immobilization occurred during 100 ms step depolarizations. Using field stimulation, Ca^2+^ release did exhibit a considerable decline from pulse to pulse during the train, also consistent with previous findings, indicating that the decline of Ca^2+^ release during a short train of APs is not correlated to modification of charge movement. Ca^2+^ currents during single or 10 Hz trains of AP‐like depolarizations were hardly detectable, were minimal during 50 Hz trains, and became more evident during 100 Hz trains in some fibers. Our results verify predictions on the behavior of the ECC machinery in response to AP‐like depolarizations and provide a direct demonstration that Ca^2+^ currents elicited by single AP‐like waveforms are negligible, but can become more prominent in some fibers during short high‐frequency train stimulation that elicits maximal isometric force.

## Graphical abstract

Bibollet et al. investigate voltage‐sensing charge movement (Q(t)), sarcoplasmic reticulum Ca^2+^ release, and Ca^2+^ ionic currents evoked by transverse tubular action potential (AP)‐like waveforms in skeletal muscle fibers. They show constant Q(t) activation but decreased Ca^2+^ release and in some fibers increasing Ca^2+^ currents during short high‐frequency bursts of AP‐like depolarizations.

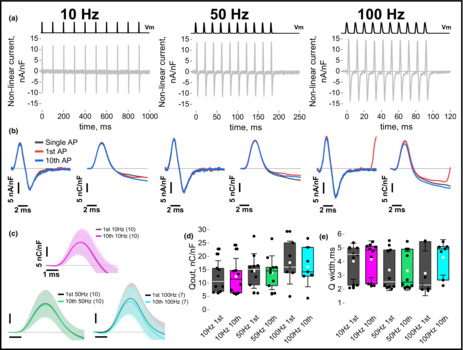

## 1 INTRODUCTION

The action potential (AP) is a self‐regenerating electrical signal that is the basis for long‐distance signaling in excitable cells such as neurons and skeletal muscle. Skeletal muscle cells are electrically polarized at rest, with an intracellular negative voltage with respect to the extracellular environment. During skeletal muscle activity, when the AP is initiated by a local depolarization at the neuromuscular junction of a skeletal muscle fiber, it propagates both longitudinally along the fiber length via the sarcolemma (surface AP) and inwardly into the fiber via the radial spread of the transverse tubule (TT) system (tubular AP), invaginations of the surface membrane (Adrian & Peachey, 1973; Gonzalez‐Serratos, 1971; Huxley & Taylor, 1958).

The AP depolarizing phase is mediated by the opening of voltage‐dependent Na^+^ channels, whereas the repolarization is due in part to both the inactivation of Na^+^ channels and the opening of voltage‐dependent K^+^ and Cl^−^ channels, with minimal influence of voltage‐dependent Ca^2+^ channels (Adrian et al., 1970; Jurkat‐Rott et al., 2006). To evoke muscle contraction, the AP modifies the conformational state of the voltage‐dependent L‐type Ca^2+^ channels (Ca_V_1.1) within the TT membrane (Rios & Brum, 1987; Schneider & Chandler, 1973).

In skeletal muscle Ca_V_1.1 channels gate two Ca^2+^ flux pathways. Ca_V_1.1 itself forms a voltage‐dependent Ca^2+^ conductance, which mediates measurable inward Ca^2+^ current across the TT membrane when using long depolarizing pulses (Beam & Knudson, 1988; Sanchez & Stefani, 1978; Stanfield, 1977). Yet, because this Ca^2+^ current is relatively small and slowly activating, numerous reports indicate that Ca_V_1.1‐mediated Ca^2+^ current may play a marginal role or no role in excitation‐contraction coupling (Bannister & Beam, 2013; Dayal et al., 2017; Lee et al., 2015).

The primary function of Ca_V_1.1 is to work as the voltage sensor (Rios & Brum, 1987; Schneider & Chandler, 1973) that regulates the voltage‐dependent opening of the ryanodine receptor type 1 (RyR1) Ca^2+^ release channels within the sarcoplasmic reticulum (SR) membrane (Imagawa et al., 1987; Meissner et al., 1986), which is abutted to, but distinct from the TT membrane where Ca_V_1.1 is positioned. The electrical manifestation of the Ca_V_1.1 voltage sensor function is the intramembranous charge movement (Rios & Brum, 1987, Schneider & Chandler, 1973) and this signal controls the activation of RyR1 channels to produce a transient elevation of myoplasmic Ca^2+^ that ultimately triggers muscle contraction. This sequential series of events is part of a process known as the excitation‐contraction coupling (ECC) of skeletal muscle (Sandow, 1952). Yet, the molecular specifics of the mechanism of operation in ECC are still unknow.

The ECC voltage sensor charge movement of amphibian and mammalian muscle as well as the evoked Ca^2+^ transients and corresponding SR Ca^2+^ release flux has been extensively studied in isolated muscle fibers (Delbono et al., 1991; Ferreira Gregorio et al., 2017; Garcia & Schneider, 1993; Hollingworth & Marshall, 1981; Horowicz & Schneider, 1981; Jacquemond, 1997; Jacquemond et al., 1991; Kovacs et al., 1979; Melzer et al., 1984; Prosser et al., 2009a, 2009b; Royer et al., 2008; Schneider et al., 1987; Schneider & Simon, 1988; Shirokova et al., 1996; Simon et al., 1991; Simon & Beam, 1983; Simon & Schneider, 1988; Szentesi et al., 1997; Wang et al., 1999). While these studies revealed important salient features of the ECC such as the voltage dependence of charge movement and voltage‐dependent depletion of Ca^2+^ release upon sustained depolarizations (Jacquemond et al., 1991; Kovacs et al., 1979; Melzer et al., 1984; Schneider et al., 1987; Schneider & Simon, 1988; Simon & Schneider, 1988), they mainly are centered on the charge that moves and evoked Ca^2+^ transients in response to step‐like depolarizations.

Until recently (Banks et al., 2021), there have been no direct examinations of charge or Ca^2+^ currents in response to AP‐like waveforms. Understanding how the native physiological AP shapes the early steps of ECC in skeletal muscle is becoming a critical topic of interest and a prerequisite to further understand physiological and abnormal excitability (Banks et al., 2021; Koenig et al., 2018; Wang et al., 2022). Since Sandow coined for the first time the term ECC 70 years ago (Sandow, 1952), we have learned a great deal about its operational and structural details, yet, many questions remain unanswered (Beam & Bannister, 2010). For instance, it has been proposed but never demonstrated that changes in charge movement evoked by APs could account for suppressing the Ca^2+^ release seen during brief trains of APs. Similarly, the measurement of the Ca^2+^ influx accompanying an AP has been inferred indirectly from quenching experiments (Koenig et al., 2018; Robin & Allard, 2015) but never directly recorded during an AP‐like waveform. Consequently, the aim of this study was twofold: (i) to investigate whether changes in charge movement account for the suppression of Ca^2+^ release during a train of APs, and (ii) to characterize the AP‐like induced Ca^2+^ influx in mammalian muscle. Here, we evaluated the properties of the ECC‐related voltage‐sensing current, the Ca^2+^ transient and the underlying Ca^2+^ release flux that it evokes from the RyR1s, and unexpectedly, demonstrate Ca^2+^ current activation only in a fraction of the fibers studied during trains of tubular AP‐like waveforms.

## 2 MATERIALS AND METHODS

### 2.1 Animals

All the animal procedures and protocols were reviewed and approved by the Institutional Animal Care and Use Committees of the University of Maryland. Male C57BL/6J mice (Charles River, Wilmington, MA) were used. A total of 35 C57BL/6J male mice were used. All mice were between 30 and 60 days of age. Environmental conditions were maintained with a 12‐h light/dark cycle and constant temperature (21–23°C) and humidity (55 ± 10%). The cages contained corncob bedding (Harlan Teklad 7902) and environmental enrichment (cotton nestlet). Mice were supplied with dry chow (irradiated rodent diet; Harlan Teklad 2981) and water ad libitum.

### 2.2 Muscle fiber culture

Culture of flexor digitorum brevis (FDB) was carried out as previously described (Hernández‐Ochoa et al., 2012; Liu et al., 1997). FDB muscles are composed predominantly of fast type IIa and IIX fibers (Tarpey et al., 2018). Animals were euthanized by asphyxiation via CO_2_ followed by cervical dislocation. FDB muscle was isolated and enzymatically dissociated with collagenase type I (2 mg/mL; Sigma‐Aldrich) in S‐MEM (Life Technologies, Cat No. 11380037) with 10% FBS, and 50 μg/mL gentamicin for 3 h ± 15 min at 37°C in 5% CO_2_. Muscle was then gently triturated to separate fibers. Individual fibers (20–50 fibers per dish) were plated in MEM media (Life Technologies, Cat No. 11095080) with 10% FBS on glass‐bottomed dishes (Matek Cor., Cat. No. P35G‐1.0‐14‐C) coated with laminin (Thermo Fisher, Cat. No. 23017–015). Fibers were maintained in culture for 14 to 18 h at 37°C, 5% CO_2_ to facilitate attachment to substrate and studied within the first 20 h after isolation to avoid dedifferentiation effects (Brown & Schneider, 2002).

### 2.3 Two‐electrode voltage clamp (TEVC) and tubular action potential voltage clamp (APVC)

To measure non‐linear capacitive currents, Ca^2+^ currents, and Ca^2+^ transients elicited by a T tubular AP‐like waveform, we used optical measurements of propagated AP obtained with high‐speed confocal line scan imaging (Zeiss LSM5 Live; 20 μs/line or 50,000 lines/s) with the potentiometric dye Di‐8‐ANEPPS in intact non‐clamped fibers, which were activated by field stimulation. Importantly, the trajectory of di‐8‐ANNEPS line scan signal monitors voltage changes across the TT system, which is characterized by its own repertoire of ion channel and passive properties when compared to surface sarcolemma (DiFranco et al., 2005; Jurkat‐Rott et al., 2006). Because Ca_V_1.1 is more likely to experience this tubular AP‐like waveform, we favored its use in the following sections. This tubular AP signal obtained in intact fibers was averaged, digitized, and used a voltage command waveform in voltage clamp experiments utilizing TEVC as previously described (Banks et al., 2021). The AP‐like depolarization amplitude was adjusted using the gain of the amplifier to match the trajectory of the optically recorded AP. Di‐8‐ANEPPS signals were scaled applying a calibration of 0.08–0.13 ΔF/F0 ~ 100 mV (DiFranco et al., 2005). Only short FDB fibers (<500 μm in length and 20–45 μm in width) were chosen and visualized on a Zeiss Axiovert 200 M inverted microscope. The external recording solution composition was low‐Cl^−^ and Na^+^‐free (in mM): 150 TEA‐CH_3_SO_3_, 10 HEPES, 1 MgCl_2_, 0.001 tetrodotoxin (TTX), 0.5 4‐aminopiridine (4‐AP), 0.025 BTS (N‐benzyl‐p‐toluene sulphonamide), pH adjusted to 7.4 with CsOH, osmolarity of 295 mOsm. Where noted, 0.5 mM Ca^2+^ plus 1.5 mM Cd^2+^ or 2 mM Ca^2+^ were added to the external solution to measure non‐linear currents or Ca^2+^ currents, respectively. This external solution was used for impalement. The current injecting electrode (V1) and voltage sensing electrodes (V2) were filled with a solution consisted of (in mM): 100 Cs‐CH_3_SO_3_, 20 EGTA, 6 MgCl_2_ (free [Mg^2+^] ~ 1 mM), 11.5 CaCl_2_ (free [Ca^2+^] ~ 100 nM), 5 ATP disodium, 5 phosphocreatine disodium, 10 HEPES, pH 7.4 (CsOH); osmolarity of 314 mOsm. Microelectrode V1 was placed at the middle of the selected fiber, and V2 was positioned halfway between the middle and the end of the selected fiber. This arrangement allows for symmetrical current injection via V1, and V2 measures average Vm between the middle and end of the fiber.

We used an AxoClamp 900A TEVC amplifier and Axon™ Digidata® 1550B low‐noise digitizer (Molecular Devices), HS‐9A x1 headstages were used for V1 and V2, and borosilicate glass (Warner Instruments, Cat No. G150TF‐3). The resistance of the current injecting electrode was 3–8 MΩ and 10–15 MΩ for the V‐sensing electrode when filled with this internal solution. Once the fibers were impaled with both microelectrodes, cells were held at −80 mV. Fibers with signs of clamp error, such as unstable holding current (or >−20 nA) or rapid drifts on holding potential, were rejected from the analysis. Measurements started 10 minutes after TEVC clamp configuration was established. Voltage protocols were generated, and current responses were digitized and stored in Clampex and Clampfit (version 11, Molecular Devices). Command pulses were delivered at 15 s intervals to the levels and duration indicated in each figure from a holding potential of −80 mV, unless otherwise indicated. Currents were typically low‐pass‐filtered at 3–10 kHz (3‐pole Bessel filter). Currents were sampled at 50 kHz. Linear capacitive and ionic currents were routinely subtracted by a P/−4 protocol (Armstrong & Bezanilla, 1974). The adequacy of the P/−4 protocol to eliminate linear components is shown in Supporting Information Figure S1. Charge moving out during the depolarization phase of the AP (Q_out_) and charge moving in (Q_in_) during AP repolarization were quantified as previously reported (Banks et al., 2021). Total charge movement was normalized to the linear fiber capacitance, which was determined by measuring linear capacitive current elicited by a 10 mV test pulses from a −120 mV prepulse and integrating the area under the linear capacitive current trace to estimate the membrane capacitance (C = Q/V). Amplifier feedback gain was increased individually for all fibers (180–220 A.U.), at a level below oscillations. Here, we restricted our voltage clamp AP waveforms to frequencies between 10 and 100 Hz. AP frequencies above 100 Hz were not assessed mostly because 100 Hz elicited maximal force, and due to limitations with the voltage control when working with TEVC.

### 2.4 Fluorescent probes and channel blockers

We used Fluo‐4 acetoxymethyl (fluo‐4 am), a membrane‐permeable non‐ratiometric high affinity Ca^2+^ indicator (Thermo Fisher, Cat. No. F14201), and 4‐[2‐(6‐(dioctylamino)‐2‐naphthalenyl) ethenyl]‐1‐(3‐sulfopropyl)‐, inner salt (di‐8‐ANEPPS), a membrane‐impermeable potentiometric dye (Thermo Fisher, Cat. No. D3167). Fluo‐4 am and di‐8‐ANNEPS were dissolved in Pluronic F‐127 (Thermo Fisher, Cat. No. P6867) 2.5% solution in DMSO. Stock solutions were prepared for TTX (1 mM in H2O; Sigma‐Aldrich, Cat No. 554412), 4‐AP (1 M in ethanol; Sigma‐Aldrich, Cat No. 275875), CdCl2 (1 M in H2O; Sigma‐Aldrich, Cat No. 208299), and BTS (50 mM in DMSO; Sigma‐Aldrich, Cat No. 203895). Drugs and toxins were added to the TEA‐based or Ringer's solution from the stock solutions. The final DMSO and ethanol concentrations were 1:1000 or 1:2000.

### 2.5 Fiber staining/loading

Fiber loading with fluo‐4 and subsequent imaging and analysis were performed as previously described (Prosser et al., 2008; Robison et al., 2017) but with some modifications. Briefly, cultured FDB fibers were loaded with fluo‐4 am (2 μM for 60 min at 22°C) in 1 mL of L‐15 media (ionic composition in mM: 137 NaCl, 5.7 KCl, 1.26 CaCl_2_, 1.8 MgCl_2_, pH 7.4; Life Technologies, Cat No. 21083027) supplemented with 0.25% w/v bovine serum albumin (BSA; Jackson ImmunoResearch, Cat. No. 001–000‐161). Then, the fibers were washed thoroughly with plain L‐15 media to remove residual fluorescent dye. Di‐8‐ANEPPS dye staining and AP recordings were performed as previously described (Banks et al., 2018; Hernandez‐Ochoa et al., 2013, 2015; Prosser et al., 2010). FDB fibers were stained with 2.5 μM di‐8‐ANEPPS in the incubator for 3 h, followed by 2–3 washes in L‐15 media before imaging. Where noted, BTS (10–50 μM) was added to the recording solution to minimize contractile responses (Bruton et al., 2006). All single fiber recordings utilizing field stimulation were performed at room temperature in modified Ringer's solution (ionic composition in mM: 140 NaCl, 4 KCl, 2 CaCl_2_, 1 MgCl_2_, 5 D‐glucose, 10 HEPES, pH 7.4 adjusted with NaOH, osmolarity of 305 mOsm). Confocal imaging of fluo‐4 (20 μs/line) and di‐8‐ANEPPS (20 μs/line) were carried out independently using high‐speed confocal system LSM 5 Live system (Carl Zeiss). Fluo‐4 was excited with a 488 nm laser, and the fluorescence emitted >505 mm was detected. Di‐8‐ANEPPS stained fibers were excited with a 532 nm laser; emitted light was collected with a 550 nm LP filter. Fibers stained or loaded with di‐8‐ANEPPS or fluo‐4, respectively, were viewed on a Zeiss Axiovert 200 M inverted microscope and confocal imaging was performed in line scan xt mode as previously described (Banks et al., 2018; Hernandez‐Ochoa et al., 2013, 2015), with images acquired for 0.4 to 1.5 s, using a 60x (1.2 N.A.) water immersion objective giving a x‐y resolution of 0.20 μm/pixel and axial resolution of 0.77 μm.

### 2.6 Field stimulation

Electrical field stimuli were applied via two parallel platinum wires closely spaced (5 mm). Application of each stimulation protocol was synchronized relative to the start of fluorescence acquisition. Each stimulating pulse (0.5 ms, 20 V/cm) was delivered alternating the polarity using a custom‐made pulse generator. Only fibers responding with action potential‐induced twitch responses for both polarity stimulation before and after staining were studied (Hernandez‐Ochoa et al., 2016). Typically, the field stimuli were applied 70 ms after the start of the recording confocal scan sequence, thus providing control images before stimulation at the start of each sequence. These control image segments were used to determine the resting steady‐state fluorescence level (*F*_0_). Average intensity of fluorescence within the selected optical field was recorded and background corrected by subtracting an average value recorded outside the cell. The average *F*_0_ value in each trace before electrical stimulation was used to scale fluorescent signals (fluo‐4, di‐8‐ANEPPS) in the same field as Δ*F*/*F*_0_ or −Δ*F*/*F*_0_. Line scan was positioned perpendicular to the main axis of the fiber, as close as possible to its center. Region of interest (ROI) used to obtain *F* vs. time profiles was placed in the center of the line scan with a × dimension of 42 pixels at 0.20 μm/pixel.

### 2.7 Calculation of SR Ca^2+^ release flux from intact fibers using fluo‐4 fluorescence recordings

Fluo‐4 fluorescence recordings (F) were used to estimate the time course of the free [Ca^2+^] as previously described (Prosser et al., 2010; Yamaguchi et al., 2011). A Ca^2+^ removal model including binding and transport was used to estimate the time course of Ca^2+^ release flux during action potential‐induced activation (Baylor & Hollingworth, 2003; Melzer et al., 1987). Binding to Ca^2+^‐specific sites of troponin C (T‐sites) and parvalbumin‐like Ca^2+^‐Mg^2+^ sites (P‐sites), as well as fast Ca^2+^ binding to ATP was calculated using binding site concentrations, rate constants, and approximations adopted from previous reports (Baylor & Hollingworth, 2003; Prosser et al., 2010; Yamaguchi et al., 2011). The fixed rate constant values used for the calculations were for T‐sites: kon, T, Ca = 115 μM/sec, koff, T, Ca = 150/sec; and for P‐sites: kon, P, Ca = 54.0 μM/sec, koff, P, Ca = 0.65/sec, kon, P, Mg = 0.043 μM/sec, koff, P, Mg = 3.9/s, kuptake = 1000/s. [T]tot and [P]tot, the total concentrations of T‐sites and P‐sites, were 0.240 mmol/L and 1.5 mmol/L, respectively. The Ca^2+^ occupancies of all model compartments [T‐sites, P‐sites, ATP (F‐sites), and uptake] were summed, and the Ca^2+^ release flux was calculated as the time derivative of the sum (Timmer et al., 1998). Calculations were performed using Euler's method (Scarborough, 1966). Analysis was performed using Excel Solver (Microsoft).

### 2.8 Data analysis

Data spreadsheets were generated from the raw fluorescent images. Data were initially processed in Excel (Microsoft). Visual Basic (Microsoft) macros were used to systematically determine the Δ*F*/*F*_0_ or −Δ*F*/*F*_0_ values. Data were then analyzed and plotted using OriginPro 2021 (OriginLab Corporation). *F* (t) records from di‐8‐ANEPPS and fluo‐4 were acquired at 50 KHz and were smoothed using adjacent‐averaging function with a 25 points window. The precision and synchronization between the LSM 5 Live confocal and voltage clamp amplifier was corroborated as previously reported (Banks et al., 2021).

### 2.9 Muscle fiber membrane current analysis

Data analysis was performed using Clampfit 8.0 (Molecular Devices). Further data evaluation, non‐linear fitting, and statistical analysis were conducted using OriginPro 2021 software. Q‐t relationship of each individual fiber was obtained by integration of the capacitive current from *t* = 0 to *t* = 10–30 ms elicited by single AP or repetitive AP waveforms. Width of both non‐linear current and charge was quantified as the width at 50% of its maximum amplitude. Q_out_ was quantified by integration of the non‐linear current signal, beginning at the initiation of the depolarization, passing by the maximum outward part of the signal and ending when non‐linear current was equal to zero, just before becoming a negative value. Q_in_ was evaluated from the running integral, from its maximum to the time when it crossed the *x*‐axis.

### 2.10 Assay of FDB function in vivo

FDB in vivo force measurement was assessed as previously described (Geist et al., 2018). Briefly, in isoflurane‐anesthetized mice, the hindfoot was immobilized and the toe was secured to a force transducer with silk suture around the pad of the fourth digit. For this assay, we use two 28 gauge monopolar electrodes (J05 Needle Electrode Needles, 36BTP, Jari Electrode Supply) placed subcutaneously ~3 mm apart just proximal to the ankle. The fourth toe was secured to the force transducer with a loop of 3–0 braided silk suture around the pad of the distal digit (3rd) to achieve maximal mechanical advantage. Brief trains (200 ms) of pulses (10–150 Hz) or single pulse depolarization through percutaneous nerve stimulation across the ankle was used to elicit isometric contractions. For each experiments, the angle of FDB extension was adapted to achieve maximal isometric force.

### 2.11 Statistical analysis and randomization

To avoid one source of systematic bias, experimental and control measurements were alternated in a randomized manner. Experimenters were not blinded to the source of the cells or recording conditions evaluated. Normal distribution of data was assessed using the Kolmogorov–Smirnov test. Summary data were reported as mean ± SD. Summary plots displayed as box and whiskers show mean (white square), standard deviation (whisker), median (horizontal line within the box), 1st and 3rd quartile (box delimitation), and individual data (black dots). Statistical significance was assessed using either parametric two sample *t*‐test, pair sampled t‐test, or one‐way ANOVA or one‐way repeated measures ANOVA followed by Scheffe post hoc comparison. The type I error was set *α* = 0.05, and Origin version 9.9 (OriginLab Corporation) was used through the analysis.

## 3 RESULTS

### 3.1 Non‐linear current, charge movement, and Ca^2+^ current during a single AP‐like waveform

**FIGURE 1:**
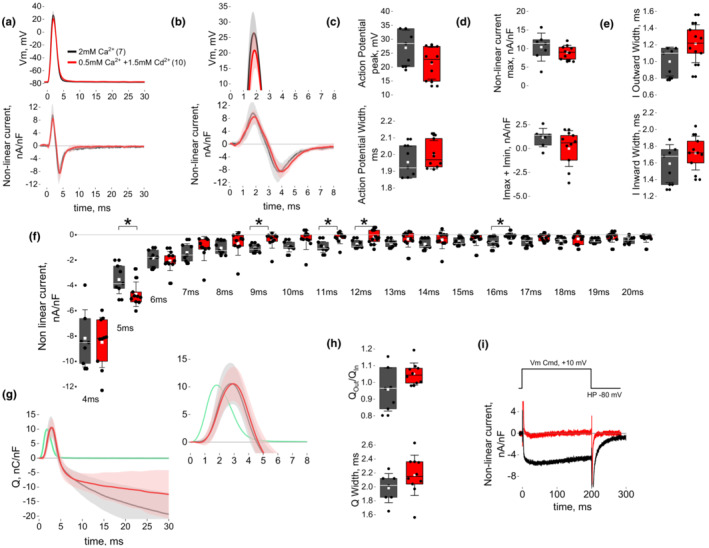
Single tubular AP‐like waveform evokes charge movement and negligible Ca^2+^ current. (a) Average voltage response acquired by imposing a tubular AP‐like voltage clamp waveform in FDB fibers in presence of 2 mM Ca^2+^ (*N* = 3, *n* = 7, black) or 0.5 mM Ca^2+^ + 1.5 mM Cd^2+^ (*N* = 3, *n* = 10, red) in the external solution (top) and corresponding non‐linear current relative to membrane capacitance vs. time (bottom). (b) Zoom‐in to the maximal voltage response (top) and of the non‐linear current (bottom) form data presented in a. (c) Maximal voltage response (top, Ca^2+^ = 26.94 ± 6.35 vs. Cd^2+^ = 21.13 ± 6.00 mV, *p* = 0.08) and voltage width (bottom, Ca^2+^ = 1.95 ± 0.10 vs. Cd^2+^ = 2.00 ± 0.09 ms, *p* = 0.29) show similar AP‐like depolarization for Ca^2+^ vs. Ca^2+^ + Cd^2+^ conditions. (d) Maximal non‐linear outward current response (top, Ca^2+^ current = 10.39 ± 3.78 vs. Cd^2+^ = 8.97 ± 1.89 nA/nF, *p* = 0.321) and addition of the maximum non‐linear outward current and the minimum inward non‐linear current (bottom, Ca^2+^ = 1.15 ± 0.97 vs. Cd^2+^ = −0.00 ± 1.87 nA/nF, *p* = 0.159) reveal no differences in non‐linear current amplitude responses obtained in Ca^2+^ vs. Ca^2+^ + Cd^2+^. (e) Outward non‐linear current width (top, Ca^2+^ = 1.00 ± 0.17 vs. Cd^2+^ = 1.21 ± 0.23 ms, *p* = 0.053) and inward non‐linear current width (bottom, Ca^2+^ = 1.59 ± 0.23 vs. Cd^2+^ = 1.79 ± 0.20 ms, *p* = 0.245) revel no differences in kinetic responses between records obtained in Ca^2+^ vs. Ca^2+^ + Cd^2+^. (f) Isochronal non‐linear current density evaluated at 1 ms intervals post stimulation reveals minimal Ca^2+^ current during a single tubular AP‐like waveform (see Table 1 for values). (g) Running integral from recordings presented in D showing charge (Q) vs. time. Note the tight overlap of the curves from the two conditions between 0 to 8 ms post stimulation. Green traces show measured AP‐like depolarization. (h) Q_out_/Q_in_ ratio (top, Ca^2+^ = 0.96 ± 0.13 vs. Cd^2+^ = 1.05 ± 0.07, *p* = 0.07) and Q width (bottom, Ca^2+^ = 1.98 ± 0.21 vs. Cd^2+^ = 2.16 ± 0.29 ms, *p* = 0.173) reveal no differences in charge amplitude and kinetics between records obtained in Ca^2+^ vs. Ca^2+^ + Cd^2+^. (i) Representative non‐linear current density as a function of the time (bottom) elicited by a 200 ms step‐like depolarization to +10 mV (top) in 2 mM Ca^2+^ (black trace) or in the presence of 1.5 mM Cd^2+^ (red trace). All data are represented as mean ± SD through brackets or light‐shadowed colors; statistical analysis was conducted using an unpaired sample *t*‐test. * denotes *p* < 0.5; see Table 1.

Using step‐like depolarizations, it has been shown that Ca_V_1.1 functions as a Ca^2+^ ion channel, but because of its slow activation kinetics its contribution to ECC appears to be irrelevant (Dayal et al., 2017). Whether the Ca^2+^ current carried by Ca_V_1.1 even becomes detectable during a single action potential (AP) in skeletal muscle has not been previously explored. In neurons and cardiac cells, Ca^2+^ currents are not measurable during the rising phase of the AP. Instead, it is during the falling phase of the AP when the Ca^2+^ current (deactivation) can be measured (Bean, 2007; Llinas et al., 1981; Ramos‐Franco et al., 2016). Here, first we sought to determine whether Ca^2+^ current is evoked by an AP‐like waveform. The AP‐like waveform was generated by measuring optically the AP using the voltage sensitive dye di‐8‐ANEPPS in unclamped muscle fibers in response to field stimulation, which initiates a propagated AP in the surface and transverse tubules. This di‐8‐ANEPPS signal was digitized and used as a template to generate an AP‐like voltage‐clamp waveform that followed the trajectory of the tubular AP measured optically (Banks et al., 2021). This approach was chosen over conventional AP‐voltage clamp, which uses current‐clamp to first initiate and measure the AP and then uses this recorded AP as voltage command, to avoid the depolarization threshold “shoulder” commonly observed in current‐clamp experiments (DiFranco et al., 2005; Manno et al., 2013). Next, to determine whether Ca^2+^ current is evoked in response to tubular AP‐like waveform, we used standard recording solutions designed to suppress Na^+^, K^+^, and Cl^−^ currents. We measured independently non‐linear membrane currents without and with the Ca^2+^ channel blocker Cd^2+^ to be able to isolate Ca^2+^ currents and to visualize non‐linear capacitive currents plus any unblocked residual non‐linear ionic currents, respectively, during an imposed AP‐like waveform. For these experiments, the Ca^2+^ channel blocker nifedipine was not used due to its additional effects on charge movement and SR release (Rios & Brum, 1987). Instead, we used Cd^2+^ at a concentration known to block Ca_V_1.1 L‐type Ca^2+^ currents in skeletal muscle fibers (Robin & Allard, 2015). Figure 1a,b (upper traces) shows the recorded membrane potential in response to tubular AP‐like command and the evoked non‐linear current (Figure 1a,b, lower traces) in control (2 mM Ca^2+^, black) or in the presence Cd^2+^ (1.5 mM plus 0.5 mM Ca^2+^, red) to suppress the Ca^2+^ currents. Note that this tubular AP‐like depolarization tracks the averaged signal obtained from tubular di‐8‐ANEPPS measurements in multiple fibers (Banks et al., 2021). The tubular AP‐like command's amplitude and width were similar with and without Ca^2+^ channel blocker Cd^2+^ (Figure 1c). The amplitude and width of the non‐linear current moving outward and inward in response to the AP waveform were similar whether or not the Ca^2+^ channel blocker Cd^2+^ was present in the recording chamber (Figure 1d,e). A marginal but significant difference in the non‐linear inward current was observed at 5, 9, 11, 12, and 16 ms, due likely to intrinsic noise, but no differences were observed for the other time intervals (Figure 1f and Table 1).

**TABLE 1:** Non‐linear current evaluated with 1 ms time increments reveals little Ca^2+^ ionic current induced by a single AP‐like waveform.

Time	Condition	I non‐linear, nA/nF mean ± SD	*p* value Ca^2+^ vs Cd^2+^
0 ms	2 mM Ca^2+^	0.06 ± 0.16	0.18475
	0.5 mM Ca^2+^ + 1.5 mM Cd^2+^	0.22 ± 0.28	
1 ms	2 mM Ca^2+^	2.03 ± 1.43	0.55786
	0.5 mM Ca^2+^ + 1.5 mM Cd^2+^	2.6 ± 2.38	
2 ms	2 mM Ca^2+^	8.36 ± 2.76	0.84273
	0.5 mM Ca^2+^ + 1.5 mM Cd^2+^	8.13 ± 1.94	
3 ms	2 mM Ca^2+^	−2.07 ± 3.27	0.24178
	0.5 mM Ca^2+^ + 1.5 mM Cd^2+^	−0.60 ± 1.69	
4 ms	2 mM Ca^2+^	−8.18 ± 2.26	0.75972
	0.5 mM Ca^2+^ + 1.5 mM Cd^2+^	−8.50 ± 1.98	
5 ms	2 mM Ca^2+^	−3.54 ± 1.11	**0.03714**
	0.5 mM Ca^2+^ + 1.5 mM Cd^2+^	−4.70 ± 0.97	
6 ms	2 mM Ca^2+^	−1.83 ± 0.65	0.49429
	0.5 mM Ca^2+^ + 1.5 mM Cd^2+^	−2.08 ± 0.75	
7 ms	2 mM Ca^2+^	−1.41 ± 0.71	0.46208
	0.5 mM Ca^2+^ + 1.5 mM Cd^2+^	−1.09 ± 0.96	
8 ms	2 mM Ca^2+^	−1.05 ± 0.51	0.35303
	0.5 mM Ca^2+^ + 1.5 mM Cd^2+^	−0.68 ± 0.94	
9 ms	2 mM Ca^2+^	−0.42 ± 0.31	**0.02714**
	0.5 mM Ca^2+^ + 1.5 mM Cd^2+^	−0.42 ± 0.64	
10 ms	2 mM Ca^2+^	−0.95 ± 0.40	0.10367
	0.5 mM Ca^2+^ + 1.5 mM Cd^2+^	−0.41 ± 0.75	
11 ms	2 mM Ca^2+^	−0.97 ± 0.44	**0.00546**
	0.5 mM Ca^2+^ + 1.5 mM Cd^2+^	−0.24 ± 0.46	
12 ms	2 mM Ca^2+^	−0.75 ± 0.31	**0.03664**
	0.5 mM Ca^2+^ + 1.5 mM Cd^2+^	−0.22 ± 0.55	
13 ms	2 mM Ca^2+^	−0.67 ± 0.28	0.21435
	0.5 mM Ca^2+^ + 1.5 mM Cd^2+^	−0.33 ± 0.63	
14 ms	2 mM Ca^2+^	−0.75 ± 0.40	0.24677
	0.5 mM Ca^2+^ + 1.5 mM Cd^2+^	−0.39 ± 0.72	
15 ms	2 mM Ca^2+^	−0.55 ± 0.30	0.32163
	0.5 mM Ca^2+^ + 1.5 mM Cd^2+^	−0.32 ± 0.52	
16 ms	2 mM Ca^2+^	−0.60 ± 0.41	**0.00885**
	0.5 mM Ca^2+^ + 1.5 mM Cd^2+^	−0.11 ± 0.26	
17 ms	2 mM Ca^2+^	−0.52 ± 0.35	0.06328
	0.5 mM Ca^2+^ + 1.5 mM Cd^2+^	−0.22 ± 0.27	
18 ms	2 mM Ca^2+^	−0.47 ± 0.33	0.46209
	0.5 mM Ca^2+^ + 1.5 mM Cd^2+^	−0.32 ± 0.46	
19 ms	2 mM Ca^2+^	−0.50 ± 0.19	0.07748
	0.5 mM Ca^2+^ + 1.5 mM Cd^2+^	−0.19 ± 0.40	
20 ms	2 mM Ca^2+^	−0.41 ± 0.27	0.39691
	0.5 mM Ca^2+^ + 1.5 mM Cd^2+^	−0.28 ± 0.32	
21 ms	2 mM Ca^2+^	−0.39 ± 0.29	0.18848
	0.5 mM Ca^2+^ + 1.5 mM Cd^2+^	−0.16 ± 0.35	

Notes: Data from Figure 1f. Comparison of current between Ca^2+^ and Ca^2+^ + Cd^2+^ at different time intervals was assessed by a two‐sample *t*‐test. Data represent mean ± SD. Shading and bold values indicate to identify the significant values.

We next investigated the early part of the non‐linear current corresponding predominantly to movement of voltage sensor charge arising from Ca_V_1.1 (Caputo & Bolanos, 1989; Rios & Pizarro, 1991). Figure 1g shows the time integral (Q(t)) of the non‐linear current during and after AP‐like depolarization. Note that the time course and amplitude of the charge moving out (Q_out_) and in (Q_in_) in response to AP‐like waveform were similar whether or not the Ca^2+^ channel blocker Cd^2+^ was included in the recording chamber (Figure 1g,h). These results indicate that neither amplitude nor kinetics of the charge moved by an AP waveform are modified by the presence or absence of Ca^2+^ or Cd^2+^ in the recording bath. The fact that Q(t) continues to be increasingly negative after the AP reflects the presence of a very small but maintained negative non‐linear ionic current continuing after AP termination (Figure 1g). The smaller late negative Q(t) in the presence of Cd^2+^ indicates that Cd^2+^ blocks most of the small late non‐linear inward ionic current, but residual ionic current may still be present. Figure 1i shows that exemplar Ca^2+^ currents can be evoked by a longer step‐like depolarization (200 ms). Note the slow activation kinetics of the Ca_V_1.1 ionic current (>25 ms, Figure 1i). These results show that the extent of Ca^2+^ current activation during a single AP is negligible, and confirm that Ca^2+^ or Cd^2+^ divalent ions in the recording chamber do not influence charge movement induced by an AP‐like waveform.

### 3.2 Action potential properties during brief 10, 50, 100, and 150 Hz repetitive firing using field stimulation

**FIGURE 2:**
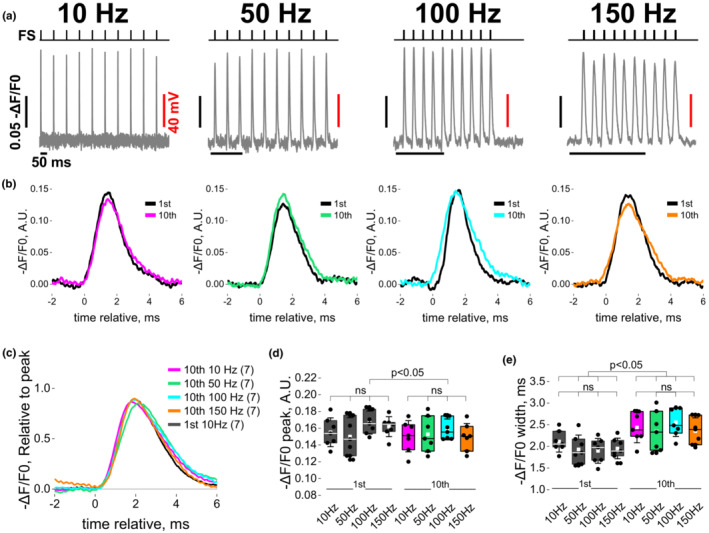
Short train of field stimulation at 10, 50, 100 and 150 Hz evokes reproducible action potentials. (a) Representative di‐8‐ANEPPS recordings elicited by external field stimulation at 10, 50, 100, or 150 Hz and corresponding zoom of the 1st and 10th depolarization within the train (b). Scale bar conversion of unit from fluorescence to mV in (a) was done assuming 0.13 − ΔF/F0 ~ 100 mV and a resting membrane potential of −80 mV, see Section 2. (c) Comparison of average ‐ΔF/F0 relative to their maximum from the 10th depolarization within the train for 10, 50, 100, and 150 Hz depolarization frequency and for the 1st depolarization within the train at 10 Hz (*N* = 3, *n* = 7). Standard deviation was not displayed to facilitate visibility. (d) Box and whisker plot of the optical AP from the 1st and 10th depolarization within the train at 10, 50, 100, or 150 Hz. A negligible but significant decrease in AP amplitude within the train was observed for 100 Hz (100 Hz 1st AP = 0.17 ± 0.01 vs. 10th AP = 0.16 ± 0.01 *p* = 3.82E‐4, pair‐sample *t*‐test). A prolongation of the optical AP width was detected at all the frequencies tested (e) (*p* = 4.73E‐5 to 0.013, pair‐sample *t*‐test). All data are represented as mean ± SD. No statistical differences were observed when comparing the response from the 1st AP at different frequencies or the 10th AP at all frequencies; one‐way repeated measure ANOVA followed by Scheffe post hoc.

In various vertebrates, including mammals, fast‐twitch muscle fibers are activated by various bursts of APs to elicit graded contraction (Hennig & Lomo, 1985; Schiaffino & Reggiani, 2011). We thus sought to evaluate the impact of repetitive APs on charge movement, Ca^2+^ release and Ca^2+^ current. We wanted to establish first whether the properties of the physiological AP change in response to repetitive stimulation in intact muscle fibers. To this end, we monitored optically APs with the membrane sensitive dye di‐8‐ANEPPS with high‐speed line scan confocal microscope, and utilized four different patterns of external field stimulation, 900 ms train at 10 Hz, 180 ms train at 50 Hz, 100 ms train at 100 Hz, and 66 ms train at 150 Hz, each of which gives 10 stimuli. These patterns of stimulation are within physiological ranges measured at the neuromuscular junctions of slow and fast twitch motor units (Hennig & Lomo, 1985). Note that recordings were done in the absence of BTS (a skeletal muscle paralyzing agent, see Section 2). Acquisition was realized in the middle of the fibers to obtain propagated tubular AP measurement and reduce movement artifact (Banks et al., 2018; Prosser et al., 2010) and in a randomized order to minimize hysteresis. Figure 2 shows that overall amplitude and kinetics of the optically measured APs in response to different frequencies of stimulation in the same fiber (Figure 2a). Zoomed in version of the 1st and 10th induced depolarization from these same fibers show that depolarizations were reproducible and similar during the train in response to all frequencies (Figure 2b). Figure 2c shows the similarities between the 1st depolarization at 10 Hz (black) and 10th depolarization at 10 Hz (green), 50 Hz (purple), 100 Hz (orange), and 150 Hz (pink), suggesting little perturbation of the induced propagated AP. When quantifying the AP's amplitude and width (Figure 2d,e), no differences were observed when comparing 1st or 10th depolarization at all frequencies, but statistically significant differences were observed when comparing 1st vs. 10th depolarization within the same frequency. These results demonstrate that for brief (<1 s) bursts of electrical activity, the amplitude of the individual propagated APs is constant while duration of the propagated tubular AP is marginally but significantly increased regardless of the frequency of depolarization.

### 3.3 Action potential evoked Ca^2+^ transients and Ca^2+^ release during repetitive firing using field stimulation

**FIGURE 3:**
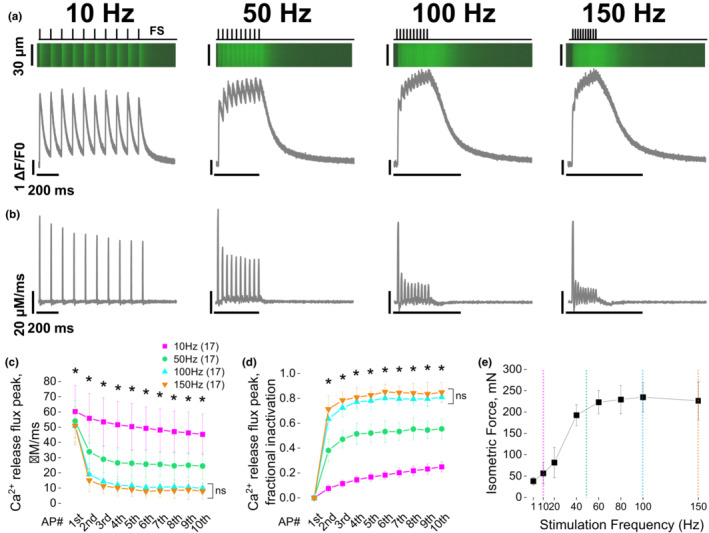
Repetitive stimulation at 10, 50, 100, and 150 Hz causes decrease of calcium release and increase of force production in a frequency‐dependent way. (a) Representative line scan confocal microscopy Ca^2+^ transients (x‐t; top) elicited by field stimulation at 10, 50, 100, or 150 Hz in FDB fibers loaded with fluo4AM and corresponding representative ΔF/F0 time courses (bottom). Top black traces indicate field stimulation depolarization. (b) Representative estimated Ca^2+^ release flux trajectories from signals presented in (a). (c) Ca^2+^ release flux peak vs. AP# within the train for 10, 50,100, and 150 Hz trains shows a Ca^2+^ release decrease that depends on the frequency of the stimulation (*N* = 3, *n* = 17, see Supporting Information Table S1). (d) Ca^2+^ release flux factional inactivation vs. the AP# within the train and the frequency of depolarization show increased inactivation during the train at all frequencies (see Supporting Information Table S1). One‐way repeated measure ANOVA followed by Scheffe post hoc revealed differences between similar AP# for all frequencies except between 100 and 150 Hz (see also Supporting Information Table S1). (e) In vivo FDB muscle force vs. frequency curve shows maximal force asymptote values at 100 Hz (*N* = 3, *n* = 3, isometric force (mN): 80 Hz = 229.33 ± 33.13, 100 Hz = 234.85 ± 34.56, 150 Hz = 226.45 ± 44.53). All data are represented as mean ± SD. * indicates *p* < 0.5, see Supporting Information Table S1.

Previous studies have demonstrated partial suppression of release flux that essentially reaches a steady state during the trains of brief (<1 s) external field stimuli (Baylor et al., 1983; Baylor & Hollingworth, 2003) or during more prolonged step depolarizations (Jacquemond et al., 1991; Melzer et al., 1984, 1986, 1987; Schneider et al., 1987; Schneider & Simon, 1988; Shirokova et al., 1996). To reassess this property, we next used field stimulation and monitored Ca^2+^ transient responses, using the non‐ratiometric dye fluo‐4, during repetitive 10, 50, 100, and 150 Hz stimulation using an high‐speed confocal imaging. Figure 3a shows representative line scan profiles and corresponding fluo‐4 transients from fibers stimulated by a train of 10 APs at 10, 50, 100, and 150 Hz in a randomized order to avoid hysteresis, with 2 minutes rest between protocols using as in Figure 2. We utilized a Ca^2+^ removal model (Melzer et al., 1987; Timmer et al., 1998; see Section 2) to calculate the time course of SR Ca^2+^ release flux from AP‐evoked fluo‐4 responses. The rate of SR Ca^2+^ release was then calculated as the time derivative of free, bound, and pumped Ca^2+^. By utilizing the same fixed and free parameters for uptake systems previously characterized (Prosser et al., 2010; Yamaguchi et al., 2011), the Ca^2+^ release flux was calculated from individual fluo‐4 fluorescence time courses of all fibers challenged with either 10, 50, 100, and 150 Hz AP trains. In Figure 3b, representative profile of the Ca^2+^ release flux can be observed for all frequencies of depolarizations. Evolution of the average SR Ca^2+^ release flux peak from the first to the last spike in the train is presented in Figure 3c. Release flux was consistently more suppressed in a frequency‐dependent manner. All frequencies demonstrated partial suppression of release flux that reaches a steady state during the trains of stimuli (Figure 3c, Supporting Information Table S1). To measure the extent of the release suppression, we divided the peak release evoked by the AP# (Rel_AP#_) by the peak release elicited by the first AP during the train (Rel_1st_). Fractional inactivation was calculated as 1 − (Rel_AP#_/Rel_1st_) (Figure 3d). Fractional inactivation was significantly different between pacing frequencies except when comparing 100 vs. 150 Hz: fractional inactivation reached ≈0.2 at 10 Hz, ≈0.5 at 50 Hz and ≈0.8 at 100 and 150 Hz (Figure 3d, Supporting Information Table S1). Next, to validate the stimulation frequencies used, we assessed whole muscle FDB in situ isometric force response as a function of various depolarization frequencies in anesthetized mice (see Section 2, Figure 3e). The force vs. frequency curve followed a sigmoidal response with an asymptote at 100 Hz. Overall, these results confirm and reproduce the properties of Ca^2+^ release flux previously found in response to physiological repetitive stimulation in amphibian and mammalian muscle fibers (Baylor et al., 1983, Baylor & Hollingworth, 2003).

### 3.4 Non‐linear currents and charge movement during trains of action potentials using TEVC

**FIGURE 4:**
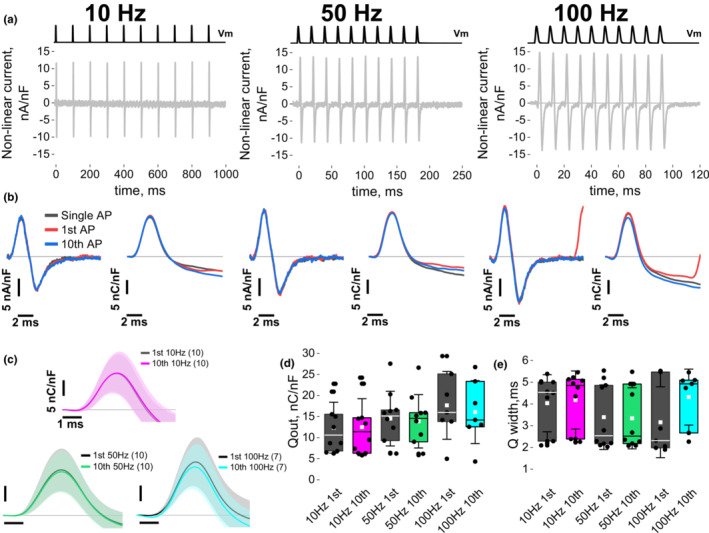
Charge movement amplitude and kinetics are not altered by tubular AP‐like trains at 10, 50, or 100 Hz. (a) Representative non‐linear current induced by 10 tubular AP‐like waveforms at 10, 50, or 100 Hz. Top black traces indicate AP‐like depolarization protocol. (b) Comparison of exemplar non‐linear currents traces vs. time induced by the 1st (red), 10th (blue) and single AP‐like waveform (black) (left) and charge running integrals Q(t) (right) for 10, 50, and 100 Hz. Note that traces in a and b are from the same recordings. (c) Average (Q‐t) from the 1st and 10th depolarization within the train elicited at 10, 50, or 100 Hz (*N* = 3–5, *n* = 7–10). (d) Summary Box and whisker plot reveal similar Q_out_ between the different frequency of depolarization (*p* = 0.30–0.57) and between the 1st and 10th depolarization within the train (*p* = 0.71–0.96), and similar Q width (e, *p* = 0.11–0.74). Data comparing signals elicited by the 1st and 10th AP in the train were assessed using a pair‐sample *t*‐test. The signals evoked by the 10th AP at different frequencies were evaluated by one‐way ANOVA followed by Scheffe post hoc. Data represent mean ± SD through brackets or light‐shadowed colors.

Having characterized the properties of the Ca^2+^ release in response to trains of APs via field stimulation, we then asked whether non‐linear currents and charge movement are also affected by repetitive AP‐like waveforms. Charge movement reduction or immobilization could, in principle, account for the suppressed Ca^2+^ release seeing during repetitive firing (Bruton et al., 2000). We measured in Figure 4 the non‐linear capacitive current as in Figure 1, but now using 10, 50, and 100 Hz trains of AP‐like depolarizations in the presence of 1.5 mM Cd^2+^. Note that 150 Hz frequency was not used since it evoked comparable force development as observed at 100 Hz (see Figure 3e). Since we showed that AP prolongation within a train is small but consistent (Figure 2), we repeated 10 times the AP waveform used in Figure 1 at the desired frequency to avoid bias of non‐linear current perturbation due to AP prolongation within the train. Figure 4a shows representative non‐linear currents evoked with 10, 50, and 100 Hz trains of AP‐like depolarizations. Figure 4b (left panels) shows a comparison of the non‐linear current elicited by a single AP‐like waveform (black), 1^st^ AP within the train (red), and 10th AP in the train (blue) for each frequency evaluated.

When doing the running integral for the first 10 ms of these signals (Q‐t, Figure 4b, traces on the right of the panels below each stimulation frequency), the initial ≈6 ms of the signals are nearly identical, while marginal differences can be seen in the following 4 ms. When we assessed the charge moved from the 1st and 10th AP‐like depolarization for multiple fibers at the three frequencies (Figure 4c), we did not observe any differences in the amount of charge moved nor kinetics (Figure 4d,e). Overall, as expected but not previously demonstrated, these results suggest that during brief trains of repeated AP‐like waveforms at physiological frequencies, the properties of the charge movement remain unchanged. Thus, a change in voltage sensor charge movement cannot account for the suppression seen in the Ca^2+^ release during short trains of APs as seen in Figure 2.

### 3.5 Non‐linear ionic currents during trains of action potentials using TEVC

We next decided to investigate non‐linear ionic current using the same approach as in Figure 4, but with 2 mM Ca^2+^ and no Cd^2+^ (i.e., no Ca^2+^ channel blocker) in the external solution. While we showed in Figure 1 that the Ca^2+^ current activation is negligible during a single AP‐like waveform, we next investigated if repetitive AP‐like activation could lead to Ca^2+^ current facilitation (Dolphin, 1996; Fleig & Penner, 1996; Garcia et al., 1990). Ca^2+^ current facilitation could make a Ca^2+^ current contribution more plausible during trains of APs (Brody et al., 1997; Dolphin, 1996).

**FIGURE 5:**
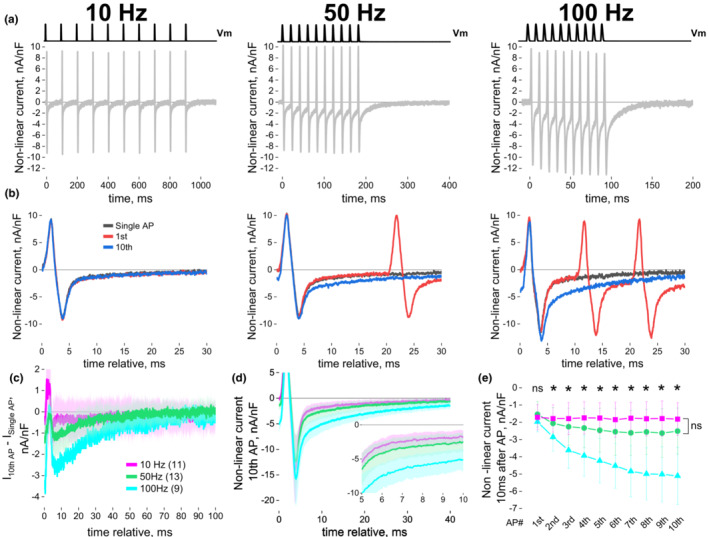
Ca^2+^ current facilitation during tubular AP‐like trains at 50 and 100 Hz but not at 10 Hz. (a) Non‐linear current evoked by tubular AP‐like waveforms at 10, 50, or 100 Hz. Top black traces indicate AP‐like waveform protocol. (b) Comparison of non‐linear current records vs. time elicited by the 1st (red), 10th (blue) and single AP‐like waveform (black) within the train; data from recording presented in (a). Note the similarities between the current profiles evoked by the 1st AP in the train and single AP response for 10, 50, ad 100 Hz, but not with that elicited by the last AP in the train (10th AP) when using 50 and 100 Hz AP‐like trains. (c) Subtraction of current elicited by the 10th AP from current evoked by a single AP reveals a potentiation of the Ca^2+^ current in a frequency‐dependent way (*N* = 3–5, *n* = 9–13). (d) Average non‐linear current initiated by the 10th AP within the train at 10, 50, or 100 Hz shows an increase in the inward current (time constant decay was: 1.37 ± 0.40 ms at 10 Hz, vs. 2.66 ± 1.74 ms at 50 Hz and 5.63 ± 2.74 ms at 100 Hz, Scheffe post hoc). (e) Non‐linear current assessed 10 ms after AP initiation shows a frequency‐dependent potentiation (see Table 2). Data comparing 1st and 10th AP within the train are paired while data representing different frequencies are from independent fibers. Data represent mean ± SD through brackets or light‐shadowed colors. Comparison of current between 1st AP and AP# was assessed by pair‐sample *t*‐test while the comparison of the same AP# at different frequencies was conducted using one‐way ANOVA followed by Scheffe post hoc * indicates *p* < 0.5.

Figure 5a shows exemplar records of a muscle fiber showing Ca^2+^ current facilitation in response to AP‐like waveforms of increasing frequency (10, 50 and 100 Hz) measured in the presence of 2 mM Ca^2+^ without Cd^2+^ For comparison, non‐linear current recordings in presence of Cd^2+^ are shown in Supporting Information Figure S2. While current responses within the train appear similar at 10 Hz, larger deactivating inward current responses emerge within the AP‐like trains at 50 Hz. In Figure 5b, non‐linear current induced by single AP (black), 1st AP within the train (red), and 10th AP within the train (blue) were lined‐up relative to the depolarization. An overlap of the single AP and 1st AP current responses can be observed at all frequencies, indicating that the 1st AP within a train would generate the same non‐linear current response as an isolated single AP depolarization. However, while the single AP and 10th AP do not show any differences at 10 Hz, the tail current induced by 10th AP is enhanced when comparing to single AP current at 50 and 100 Hz (Figure 5b). Having demonstrated that the non‐linear current does not change during a short AP‐like train at these frequencies, we subtracted the current response for the single AP from the response to the 10th AP to digitally obtain the ionic component of the recorded non‐linear current (Figure 5c). The first 6 ms of this signal shows a transient outward current due to the residual negative inward ionic current before the 10th AP depolarization. Maximal ionic current between 6 ms and around 60 ms was potentiated in a frequency‐dependent manner (≈ − 0.5 nA/nF at 10 Hz, ≈ − 1.2 nA/nF at 50 Hz, ≈ − 2.8 nA/nF at 100 Hz). We fitted the decay of the inward non‐linear current evoked by the 10th AP‐like waveform using a simple exponential, starting from maximal inward current as described before (Manno et al., 2022). The decay was faster at 10 Hz than 50 Hz but the difference was not significant (*p* = 0.233, Scheffe post hoc), while decay at 100 Hz was slower and significant when compared to 10 (*p* = 5.87E‐5 Scheffe post hoc), or 50 Hz (*p* = 0.003, Scheffe post hoc). The average decaying time constant was 1.37 ± 0.40 ms at 10 Hz, vs. 2.66 ± 1.74 ms at 50 Hz and 5.63 ± 2.74 ms at 100 Hz. Subsequently, we measured the non‐linear current 10 ms after each AP‐like depolarization to track the ionic current evolution within the train (Figure 5e). Current measurements at 10 ms were selected since they would be distant enough from non‐linear current induced by Q_in_ (Figures 1 and 4) but close to AP‐like depolarization termination and compatible with the 100 Hz depolarization frequency pattern (i.e., AP initiated every 10 ms). Non‐linear current 10 ms after 1st AP was similar for all frequencies and no potentiation was observed at 10 Hz within the train (Figure 5e, Table 2). The 50 Hz train showed a small progressive potentiation of non‐linear current at 10 ms post APs within the train (Figure 5e); the current amplitude in subsequent APs was not statistically different form the one induced by 1st AP up to the 8th AP but become significant by the end of the train (see Table 2). Upon further evaluation of a larger cohort of fibers, we were able to observe this potentiation at 100 Hz (i.e., non‐linear ionic current >2 nA 10 ms after the end of the 10th AP) but only in a subset of tested fibers (13 out of 64 fibers; Supporting Information Figure S3). In clear contrast, no ionic current potentiation was observed at any frequency when Cd^2+^ was present in the recording solution (Supporting Information Figure S2). Overall, these results suggest that during brief trains of AP‐like waveforms at physiological frequencies, the non‐linear ionic current is potentiated in a frequency‐dependent way in some but not in all the fibers examined.

**TABLE 2:** Non‐linear current evaluated with 2 mM Ca^2+^ 10 ms after each AP spike in the train reveals a frequency‐dependent ionic current potentiation.

	2 mM Ca^2+^								
	10 Hz, *n* = 11		50 Hz, *n* = 13		100 Hz, *n* = 9				
AP#	I non‐linear, nA/nF Mean ± SD	*p* value 1st AP vs AP#	I non‐linear, nA/nF Mean ± SD	*p* value 1st AP vs AP#	I non‐linear, nA/nF Mean ± SD	*p* value 1st AP vs AP#	*p* value AP# 10 Hz vs AP# 50 Hz	*p* value AP# 10 Hz vs AP# 100 Hz	*p* value AP# 50 Hz vs AP# 100 Hz
1st	−1.71 ± 0.73	N/A	−1.54 ± 0.74	N/A	−1.98 ± 0.58	N/A	ns	ns	ns
2nd	−1.78 ± 0.79	0.255	−2.07 ± 0.92	0.392	−2.85 ± 0.83	**1.84E‐05**	0.722	**0.032**	0.122
3rd	−1.79 ± 0.93	0.448	−2.26 ± 1.08	0.264	−3.62 ± 1.05	**9.60E‐06**	0.539	**0.002**	**0.017**
4th	−1.74 ± 0.93	0.703	−2.33 ± 1.14	0.062	−3.94 ± 1.18	**1.99E‐05**	0.426	**4.37E‐04**	**0.007**
5th	−1.75 ± 0.88	0.662	−2.47 ± 1.28	0.487	−4.23 ± 1.29	**2.53E‐05**	0.340	**2.28E‐04**	**0.006**
6th	−1.85 ± 1.01	0.345	−2.55 ± 1.36	0.227	−4.52 ± 1.30	**1.04E‐05**	0.394	**1.91E‐04**	**0.004**
7th	−1.76 ± 0.92	0.438	−2.61 ± 1.31	0.090	−4.84 ± 1.48	**2.03E‐05**	0.266	**2.89E‐05**	**0.001**
8th	−1.80 ± 0.99	0.447	−2.56 ± 1.31	**0.040**	−4.99 ± 1.51	**1.46E‐05**	0.462	**1.38E‐05**	**3.80E‐04**
9th	−1.78 ± 1.02	0.505	−2.63 ± 1.40	**0.004**	−5.02 ± 1.59	**3.23E‐05**	0.407	**2.59E‐05**	**8.73E‐04**
10th	−1.82 ± 0.94	0.312	−2.51 ± 1.34	**0.020**	−5.11 ± 1.65	**3.53E‐05**	0.446	**2.54E‐05**	**4.02E‐04**

Notes: Data from Figure 5e. Comparison of current between 1st AP and AP# was assessed by pair‐sample *t*‐test while the comparison of the same AP# at different frequencies was conducted using one‐way ANOVA followed by Scheffe post hoc. Data represent mean ± SD. Shading and bold values indicate to identify the significant values.

## 4 DISCUSSION

In mammalian skeletal muscle, Ca_V_1.1 operates both as a voltage sensor for ECC and as an L‐type calcium channel. Its exceptional role as voltage sensor for ECC has been unequivocally established. Yet, its role as a L‐type Ca^2+^ channel is still enigmatic (Dayal et al., 2017). Calcium currents have been systematically measured using conventional voltage clamp steps (Cognard et al., 1986; Cota & Stefani, 1984; Delbono et al., 1991; Fleig & Penner, 1996; García & Beam, 1994; Nakai et al., 1996; Prosser et al., 2009a; Robin & Allard, 2015; Stanfield, 1977; Wang et al., 1999). However, whether L‐type Ca^2+^ currents are present during single APs, either alone or within trains of APs has not been previously directly investigated in mammalian skeletal muscle.

In various excitable cells, Ca^2+^ currents are not detected during the rising phase of the AP. Instead, its activity manifests as a tail current or deactivation current, which occurs during the falling phase of the AP (Bean, 2007; Llinas et al., 1981; Ramos‐Franco et al., 2016). As expected, given the fast skeletal muscle AP and the slowly activating L‐type Ca^2+^ current, we observed little, if any, Ca^2+^ current during a single AP‐like depolarization. Likewise, using voltage clamp, trains of AP‐like voltage waveforms within a train at 10 Hz fail to recruit more Ca^2+^ current. However, a small but significant increase in the Ca^2+^ current amplitude was observed during APs within 50 Hz trains, and a more significant increase was observed during 100 Hz AP‐like trains in a subset of fibers tested. Importantly, in this study we also examined the contractile properties of FDB muscle in vivo and established that the range of frequencies we used in our in cellulo studies are within frequencies that elicit intermediate force (10–50 Hz) or maximal force (100 Hz). This observation corroborates previous findings showing that maximal force is obtained at approximately 80–150 Hz, in other predominantly fast‐twitch locomotor muscles (Iyer et al., 2016).

The increase in current amplitude during 100 Hz trains of AP‐like waveforms was sensitive to block by 1.5 mM extracellular Cd^2+^, a [Cd^2+^] level that completely blocks Ca_V_1.1 channels during longer depolarizations in skeletal muscle (Robin & Allard, 2015). However, lowering the extracellular [Ca^2+^] in the presence of Cd^+2^ could reduce the driving force for Ca^2+^, potentially reducing Ca^2+^ entry via pathways insensitive to Cd^2+^. To better characterize this Ca^2+^ influx evoked by AP‐like waveforms, a pharmacological approach using selective modulators of Ca_V_1.1 and SOCE could be used; however, the complex pharmacology of calcium channel modulators (i.e., low selectivity, use and state‐dependence: Affolter & Coronado, 1985; Hadley & Lederer, 1992; Wei‐LaPierre et al., 2022) precluded the unequivocal use of these drugs to selectively modify Ca_V_1.1 current. Using quenching of fura‐2 caused by Mn^2+^ influx, it has been shown that L‐type Ca^2+^ channels contribute to Ca^2+^ influx during long lasting depolarizations and during long trains of APs in murine skeletal muscle fibers (Robin & Allard, 2015). Our results support these observations and provide direct measurements of Ca^2+^ currents during high‐frequency physiological stimulation, which likely involves Ca^2+^ influx through Ca_V_1.1 channels.

Reduced current deactivation due to more frequent channel recruitment could explain the enhancement of the current seen with high‐frequency trains of AP‐like stimulation. In addition, facilitation of voltage‐gated Ca^2+^ current, which is a form of modulation exhibited by different members of the Ca_V_ family, could be responsible for Ca^2+^ current potentiation that we observe during 50 and 100 Hz AP trains. Several studies have shown that Ca^2+^ current facilitation can be elicited by bursts of AP waveforms, strong and brief depolarizing conditioning prepulses or repetitive step depolarizations (Brody et al., 1997; Dolphin, 1996; Fleig & Penner, 1996; Garcia et al., 1990). The electrophysiological manifestation of Ca_V_ facilitation is either augmentation of the Ca^2+^ current or faster activation kinetics in response to strong depolarizing prepulses or APs bursts (Brody et al., 1997; Fleig & Penner, 1996; Weiss et al., 2010). The term facilitation was originally defined as an increase of Ca^2+^ current resulting from prepulses to depolarizing voltages (Dolphin, 1996). Interestingly, we observed AP‐evoked Ca^2+^ current facilitation exclusively in a subset of muscle fibers. We believe that this variability could be physiologically relevant. While Ca^2+^ influx via Ca_V_1.1 channels is expendable for skeletal muscle ECC (Armstrong et al., 1972; Dayal et al., 2017), optimal refilling of the internal Ca^2+^ stores requires Ca^2+^ influx via Ca_V_1.1 to counteract SR Ca^2+^ depletion due to long lasting muscle activation (Robin & Allard, 2013, 2015). Therefore, muscle fibers endowed with Ca_V_1.1 calcium facilitation during trains of AP could maintain contractility during prolonged muscle activity and delay muscle fatigue.

Interestingly, in mouse FDB muscle, short myofibers, the type of fibers studied here, represent 94% of the muscle fiber population in the whole muscle (Tarpey et al., 2018). Suppose the Ca^2+^ current potentiation we see in vitro extrapolates in vivo to the entire muscle. If this is the case, that will imply that about 20% of the fibers in the whole FDB are endowed with this Ca^2+^ current facilitation and, therefore, could exhibit reduced or delayed fatigue. Thus, this regulation could significantly affect the overall performance of this largely fast‐twitch muscle. Currently, the mechanisms or processes underlying this observation are unknown.

One possibility to explain, at least partially, the occurrence of the AP‐evoked Ca^2+^ facilitation is the status of the SR Ca^2+^ release. For example, AP‐evoked Ca^2+^ current facilitation was not observed at 10 Hz, where the fractional Ca^2+^ release inactivation was ~20%, but were seen at 50 Hz and 100 Hz, where fractional Ca^2+^ release inactivation was ~50% and 80%, respectively. The observation that Ca^2+^ current facilitation was modulated by caffeine and ryanodine in myoballs support this idea (Fleig & Penner, 1996). Nevertheless, at this point, details about the molecular mechanism that could account for the Ca^2+^ current facilitation and its occurrence in only a subset of fibers are unknown and more work is needed to properly characterize this process.

Skeletal muscle ECC does not require Ca^2+^ influx via L‐type Ca^2+^ channels, as was early demonstrated by a pioneering study (Armstrong et al., 1972) and recently confirmed by Ca_V_1.1 Ca^2+^ current ablation studies using non‐conducting Ca_V_1.1 engineered mice (Dayal et al., 2017). A more prominent role of Ca^2+^ current is observed in cardiac cells, where the L‐type Ca^2+^ current carried by Ca_V_1.2 becomes activated during the repolarization phase of the AP and contributes to triggering Ca^2+^ induced Ca^2+^ release which is essential for cardiac ECC (Ramos‐Franco et al., 2016). Our results using trains of APs support the idea that L‐type Ca^2+^ current is negligible during ECC. However, our observation raises the possibility that the Ca^2+^ current evoked by repetitive activity could be important for other muscle functions. One possibility, in addition to its role on Ca^2+^ homeostasis and development (Chen et al., 2011; Flucher & Tuluc, 2011; Robin & Allard, 2015; Sultana et al., 2016), could be in excitation‐transcription coupling, specifically by contributing to the generation of localized subsarcolemmal and perinuclear Ca^2+^ signals (Georgiev et al., 2015). It will be interesting to evaluate whether the Ca^2+^ current facilitation we have identified becomes more frequent and/or more pronounced in muscle fibers obtained from animals subjected to forced exercise (running), or derived from differences in muscle fibers type (i.e., IIa vs. IIx, the predominant fibers types in FDB muscles, Tarpey et al., 2018), or if it is more prominent in diseased models with Ca_V_1.1 gain of function, such as myotonic dystrophy type 1 (Tang et al., 2012).

It is also possible that the Ca^2+^ current that we are recording arises from a different Ca^2+^ entry pathway, such as phasic store operated Ca^2+^ entry (pSOCE) (Koenig et al., 2018; Lilliu et al., 2020) or via TRP channels (Gailly, 2012). pSOCE is a form of ultrafast SOCE that has been detected during AP‐evoked Ca^2+^ release in mammalian muscle (Koenig et al., 2018). However, this remain unlikely since pSOCE have (1) a relatively small single channel conductance (SOCE unitary conductance: fS vs. Ca_V_1.1 pS; Hess et al., 1986; Prakriya & Lewis, 2006) making its detection by electrophysiology difficult, and (2) pSOCE activates fast in a voltage independent manner (~10 ms) but remains on for several tens of milliseconds (see Supporting Information fig. 2 in Koenig et al., 2018) and the AP‐like evoked current that we resolve fully deactivates within the first 25 ms after the stimulus. Because SOCE and Ca_V_1.1 are blocked by millimolar [Cd^2+^] (Hoth & Penner, 1993), Cd^2+^ cannot be used to separate these channels. Skeletal muscle also expresses different TRP channels (i.e., TRPC3, TRPC6, TRPV4 and TRPM4 and TRPM7) (Kruger et al., 2008; O'Neil & Heller, 2005; Vandebrouck et al., 2002). TRP channels respond to diverse stimuli, including membrane lipids, G‐proteins, Ca^2+^, heath, stretch, and redox compounds (Gailly, 2012). Like SOCE, the activation of TRPC and TRPV Ca^2+^‐permeable channels is voltage independent; therefore, its contribution during an AP could be small. TRPM4 and TRPM7 channels are expressed in muscle, yet their function has not been elucidated (Gailly, 2012). TRPM4 is Ca^2+^‐impermeable; therefore, its role to the signal we are charactering is unlikely (Vennekens & Nilius, 2007). Nevertheless, additional experiments using more selective Ca_V_1.1, SOCE and TRP agonists or blockers are needed to unequivocally identify the nature of the current detected during AP‐like depolarizations. It is possible that both Ca_V_1.1 and pSOCE‐dependent influx become activated during repetitive APs (Koenig et al., 2018) and locally participate in different aspects of Ca^2+^ homeostasis and/or Ca^2+^ signaling.

Another goal of this work was to establish whether changes in the AP and/or charge movement precede the alterations seen in Ca^2+^ release flux during repetitive stimulation. In mammalian muscle fibers, tetrads of Ca_V_1.1 are apposed by RyRs (Block et al., 1988; Franzini‐Armstrong, 2018). These RyRs are known as junctional RyRs and are thought to be under direct conformational control of the overlying Ca_V_1.1 via direct intermolecular interactions (Franzini‐Armstrong, 2018; Nakai et al., 1996; Schneider & Chandler, 1973). Numerous studies have demonstrated partial suppression of Ca^2+^ release flux during trains of stimuli (Baylor et al., 1983; Baylor & Hollingworth, 2003; Hollingworth & Baylor, 2013; Prosser et al., 2010; Yamaguchi et al., 2011) and our current results (Figure 3b–d) are consistent with these observations. This form of SR Ca release modulation is thought to provide intrinsic negative control to prevent regenerative SR Ca^2+^ release via coupled RyR1 gating or Ca^2+^ induced Ca^2+^ release (Hollingworth & Baylor, 2013). One possibility to explain this Ca^2+^ release flux suppression is via immobilization of the Ca_V_1.1's voltage sensors. Chronic depolarization promotes voltage sensors inactivation (Adrian et al., 1976; Bruton et al., 2000; Dulhunty, 1992; Rios & Pizarro, 1991). We tested whether the partial inactivation of the Ca^2+^ release correlated with changes in the triggering charge movement. Here, we found that neither the amplitude nor the kinetics of the charge movement were affected by brief trains (10, 50, or 100 Hz) of APs. However, the amount of Ca^2+^ released with subsequent APs was progressively decreased when compared to that evoked by the first AP in the train, being more pronounce when using higher frequency stimulation. Repeated activation of muscle can affect the AP features (i.e., size, propagation; Balog et al., 1994; Lannergren & Westerblad, 1986; Westerblad & Lannergren, 1986). Interestingly, our findings using optical AP measurements show that the amplitude of APs evoked by external field stimulation was predominantly unchanged during the short trains of 10 APs used in this study. A small but significant AP prolongation was observed. This subtle AP prolongation was most likely due to transient tubular ion accumulation or depletion (Almers, 1980; Cairns et al., 2004; Hostrup et al., 2021) and does not explain the progressive decay of SR Ca^2+^ release flux. It has been recently shown that APs clearly get wider during longer trains (Miranda et al., 2020; Wang et al., 2022). Widening of the AP during longer trains may increase the ionic Ca^2+^ influx we identified here. Overall, our results rule out the possibility that changes in AP properties contribute to suppressed Ca^2+^ release during a short train of APs.

SR Ca^2+^ depletion and Ca^2+^‐dependent inactivation of RyR1 are the main mechanisms that have been proposed to explain the decline of SR Ca^2+^ release flux (Baylor et al., 1983; Hollingworth & Baylor, 2013; Pape et al., 1993; Royer et al., 2008, 2010; Schneider & Simon, 1988; Shirokova et al., 1996; Simon et al., 1991). Partial suppression of the Ca^2+^ release via depletion and/or RyR1 inactivation is an important fine control mechanism. It allows the first AP to release a large amount of Ca^2+^ in order to raise the cytoplasmic Ca^2+^ as rapidly as possible to start filling the Ca^2+^ binding sites on troponin C, and then, the increment in Ca^2+^ release drops greatly to give finer control so that the exact timing and number of the subsequent APs in the train can tightly regulate the cytoplasmic [Ca^2+^] and force (Allen et al., 2008). It could also represent a safety mechanism, which presumably evolved to prevent myoplasmic [Ca^2+^] levels from exceeding the concentration required to saturate the Ca^2+^ regulatory sites on troponin, circumvents delays in fiber relaxation, and prevents unnecessary use of ATP for the re‐sequestration of Ca^2+^ while minimizing possible toxic effects that could result from extreme high levels of [Ca^2+^] (Hollingworth & Baylor, 2013). It will be interesting to assess whether more intense and repetitive activity leading to muscle fatigue results in perturbations in AP, the charge movement during APs and increased AP‐induced Ca^2+^ current facilitation.

## 5 CONCLUSION

We investigated voltage‐sensing charge movement (Q(t)), sarcoplasmic reticulum Ca^2+^ release, and Ca^2+^ ionic currents evoked by transverse tubular action potential (AP)‐like waveforms in skeletal muscle fibers. They show constant Q(t) activation but decreased Ca^2+^ release, and in some fibers, increasing Ca^2+^ currents during short high‐frequency bursts of AP‐like depolarizations. Our results confirm previous observations related to charge movement and Ca^2+^ release using step depolarizations in skeletal muscle but also provide new information about multiple early steps critical for ECC and Ca^2+^ homeostasis using more physiological stimuli, the muscle fiber AP‐like waveform. Determining these parameters using AP‐like waveforms instead of step depolarizations could be of high relevance for a fundamental understanding of skeletal muscle excitability, Ca^2+^ release, and Ca^2+^ homeostasis and establishing more accurate numerical models aiming to predict changes in ECC caused by physiological adaptations or disease conditions.

## AUTHOR CONTRIBUTIONS

H. Bibollet designed and performed most of the experiments, analyzed data, and edited the manuscript. E.L. Nguyen, D.R. Miranda A.A. Voss, and C.W. Ward designed and performed experiments, and analyzed data. E.O. Hernández‐Ochoa and M.F. Schneider conceived the project and designed the research. A. A. Voss, M.F. Schneider, and E.O. Hernández‐Ochoa wrote the paper.

## CONFLICT OF INTEREST STATEMENT

The authors declare no competing financial interests.

## ETHICS STATEMENT

All vertebrate animal experiments were performed in accordance with the Animal Welfare Act and the Office of Laboratory Animal Welfare regulations, NIH, USA. The University of Maryland Institutional Animal Care and Use Committee approved all animal protocols.

## Supporting information


Supplemental Figure S1.

Supplemental Figure S2.

Supplemental Figure S3.

Supplemental Table S1.
