# Supplementary figures and images for: Voltage sensor current, SR Ca2+ release, and Ca2+ channel current during trains of action potential‐like depolarizations of skeletal muscle fibers
Source: Physiol Rep. 2023 May 5;11(9):e15675. doi: 10.14814/phy2.15675 (PMC10163276; doi:10.14814/phy2.15675)

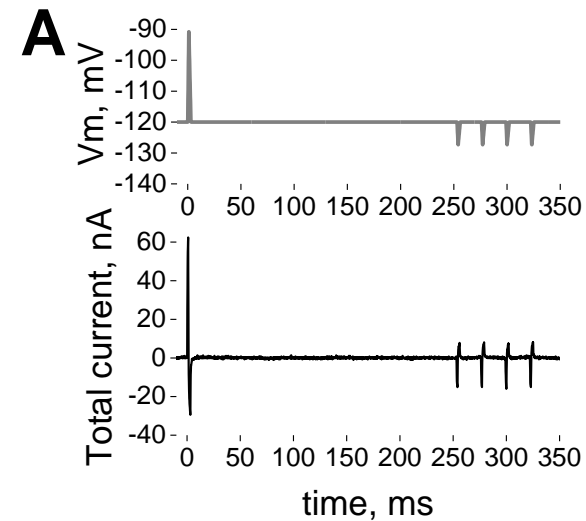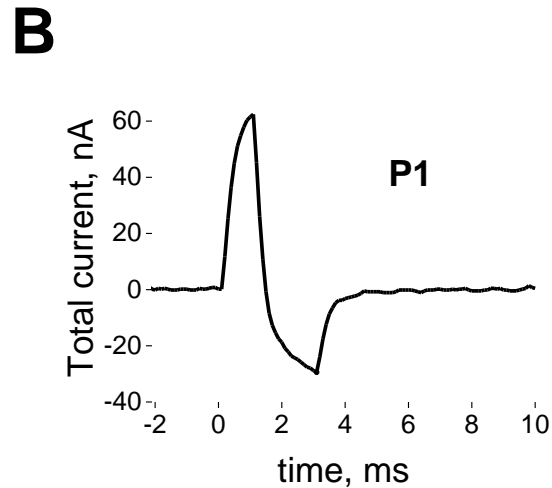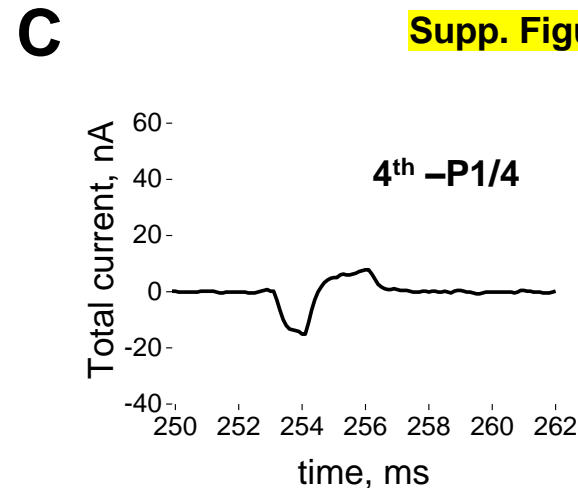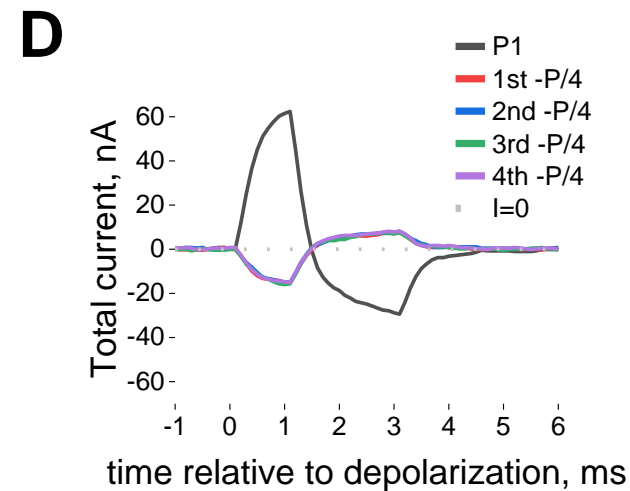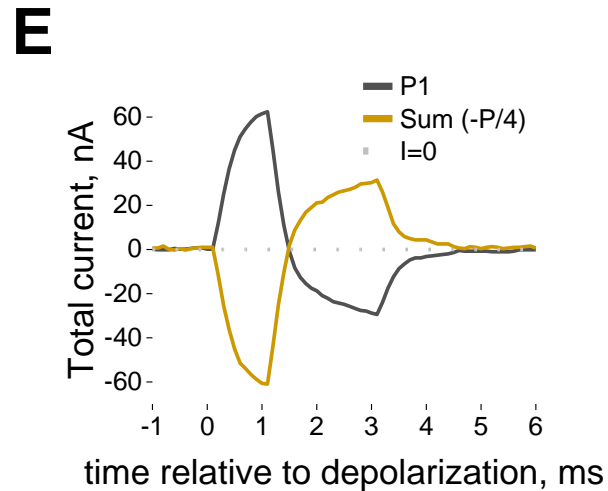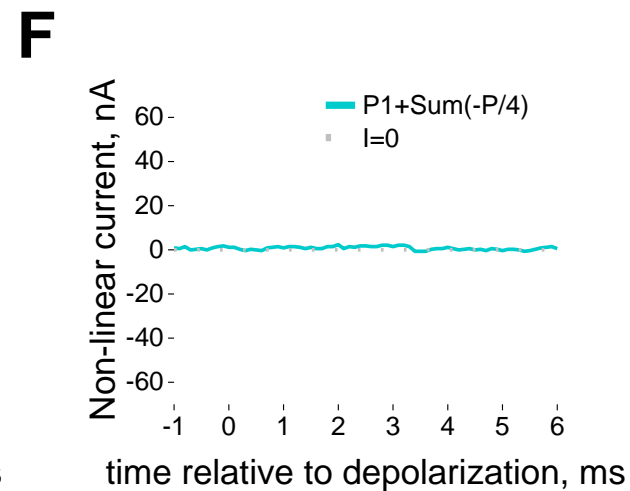

Supplement: Supplementary file 1 — Supplemental Figure S1. [file PHY2-11-e15675-s003.pdf]

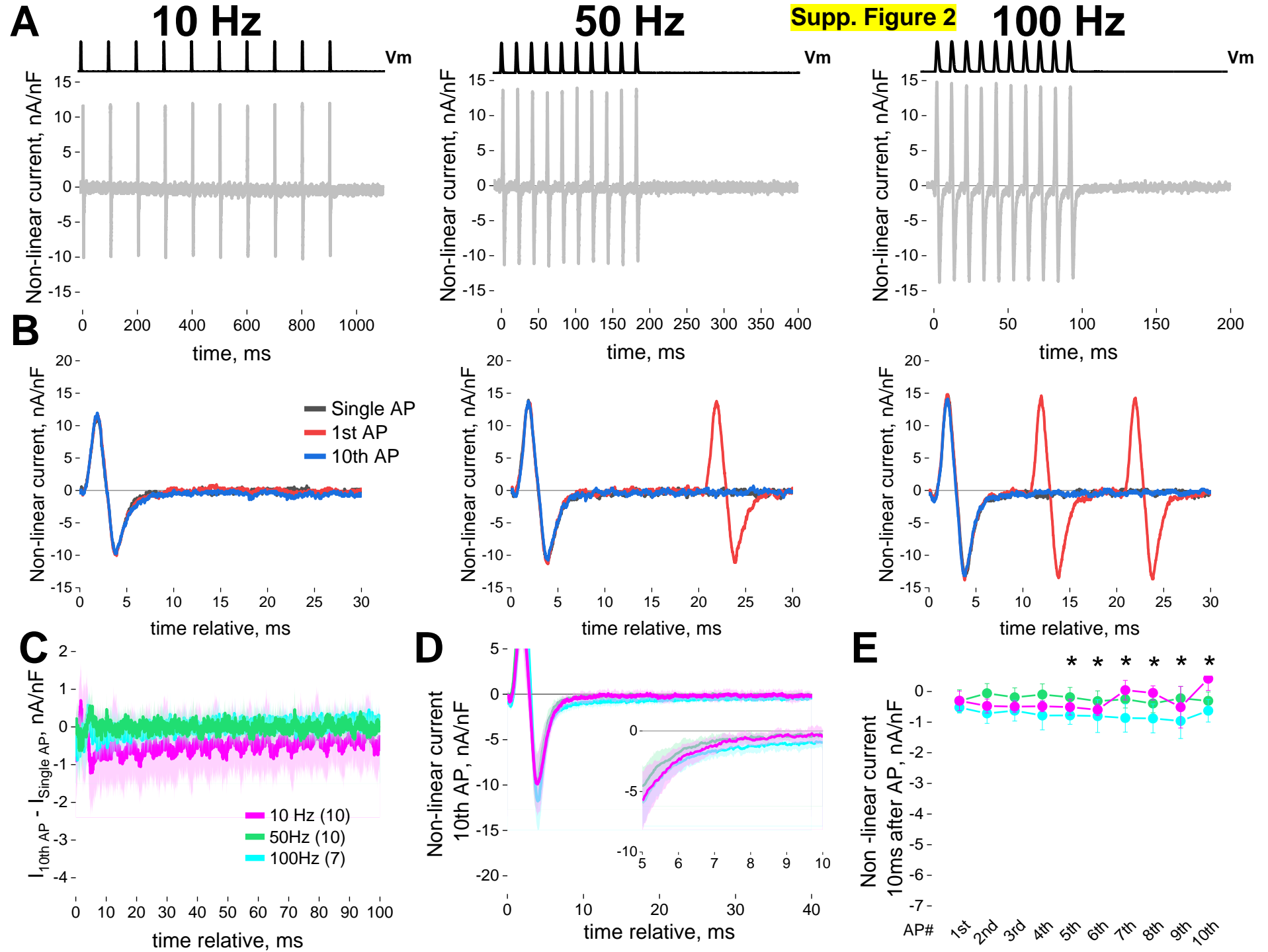

Supplement: Supplementary file 2 — Supplemental Figure S2. [file PHY2-11-e15675-s001.pdf]

**A**

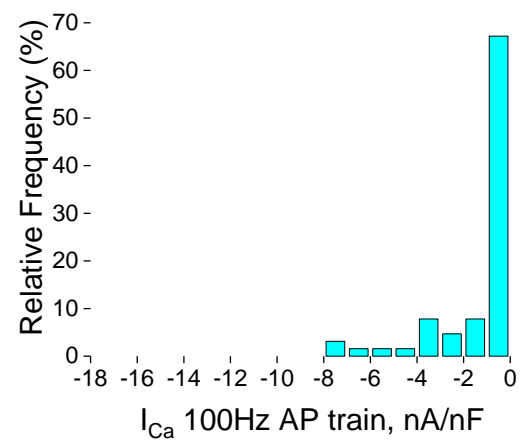

**B**

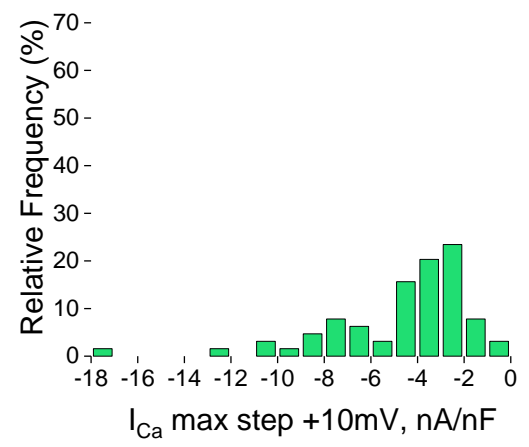

**Supp. Figure 3**

Supplement: Supplementary file 3 — Supplemental Figure S3. [file PHY2-11-e15675-s004.pdf]
