# Supplementary material for: Voltage sensor current, SR Ca2+ release, and Ca2+ channel current during trains of action potential‐like depolarizations of skeletal muscle fibers
Source: Physiol Rep. 2023 May 5;11(9):e15675. doi: 10.14814/phy2.15675 (PMC10163276; doi:10.14814/phy2.15675)
Supplement: Supplementary file 4 — Supplemental Table S1. [file PHY2-11-e15675-s002.docx]

Supp. Table 1

Supp. Table 2
